# Interconnectivity of fear of progression and generalized anxiety – Network analysis among a sample of hematological cancer survivors

**DOI:** 10.1007/s00520-023-07701-x

**Published:** 2023-03-28

**Authors:** Philipp Göbel, Katharina Kuba, Heide Götze, Anja Mehnert-Theuerkauf, Carsten Spitzer, Tim Hartung, Peter Esser

**Affiliations:** 1 Department of Medical Psychology and Medical Sociology, University Medical Center Leipzig, Leipzig, Germany; 2 Department of Psychosomatic Medicine and Psychotherapy, University Medical Center Rostock, Rostock, Germany; 3 Klinik Und Hochschulambulanz Für Neurologie, Charité-Universitätsmedizin Berlin, Berlin, Germany

**Keywords:** Fear of cancer progression, Generalized anxiety disorder, Hematologic cancer survivors, Co-occurrence, Network analysis

## Abstract

**Purpose:**

Fear of cancer progression and recurrence (FoP) and generalized anxiety disorder (GAD) are syndromes commonly seen in cancer patients. This study applied network analysis to investigate how symptoms of both concepts are interconnected.

**Methods:**

We used cross-sectional data from hematological cancer survivors. A regularized Gaussian graphical model including symptoms of FoP (FoP-Q) and GAD (GAD-7) was estimated. We investigated (i) the overall network structure and (ii) tested on pre-selected items whether both syndromes could be differentiated based on their worry content (cancer related vs. generalized). For this purpose, we applied a metric named *bridge expected influence* (BEI). Lower values mean that an item is only weakly connected with the items of the other syndrome, which can be an indication of its distinctive characteristic.

**Results:**

Out of 2001 eligible hematological cancer survivors, 922 (46%) participated. The mean age was 64 years and 53% were female. The mean partial correlation within each construct (GAD: r = .13; FoP: r = .07) was greater than between both (r = .01). BEI values among items supposed to discriminate between the constructs (e.g., *worry about many things within GAD* and *fear not to endure treatment within FoP)* were among the smallest so our assumptions were confirmed.

**Conclusions:**

Our findings based on the network analysis support the hypothesis that FoP and GAD are different concepts within oncology. Our exploratory data needs to be validated in future longitudinal studies.

**Supplementary Information:**

The online version contains supplementary material available at 10.1007/s00520-023-07701-x.

## Introduction

Anxiety appears to be a normal response in cancer patients and may even be adaptive [1–3]. When it results in disproportionately elevated levels that are not resolved over time, it can lead to severe functional impairment and may be considered a clinically relevant phenomenon [4]. Studies show that the prevalence of anxiety disorder as defined in the DSM-IV or ICD-10 in cancer patients may reach up to 19.3% [5, 6]. Among those, generalized anxiety disorder (GAD) is relevant for cancer patients and should be screened for according to clinical guidelines [1]. GAD is characterized by generalized and persistent anxiety, worries and fear, mostly related to events, situations or problems in daily life [7, 8]. The applicability of GAD among cancer patients has been discussed. Pathological fears are characterized by irrational beliefs, whereas cancer presents a real object of concern [9]. Therefore, two concepts have been proposed: fear of progression (FoP) and fear of cancer recurrence (FCR). Both focus on fear of progression or recurrence of the disease, whereas FoP applies not only to cancer but to a variety of diseases (cancer, diabetes mellitus, rheumatoid arthritis) [9]. With regard to cancer, both concepts are conceptually similar and we have used the term FoP in the following way [10]: it comprises fears related to cancer-specific aspects such as follow-up examinations, invasive therapies or pain and is conceptualized as a continuum ranging from functional to dysfunctional [11, 12]. FoP is associated with poorer psychological well-being and increased health care costs [13–15]. There is a lengthy ongoing debate about the interrelationship between FoP and GAD [16]. Various studies have shown that there were proportions of cancer patients who reported clinically relevant levels of FoP and met the criteria for GAD or another anxiety disorder, whereas others did not (13.4—61%) [16–18]. It is unclear whether there is true comorbidity or whether FoP is an epiphenomenon of an anxiety disorder [16]. It is therefore necessary to gain a better understanding about core differences and commonalities between FoP and GAD. A recent transdiagnostic FoP model emphasizes the importance of intrusive thoughts, death anxiety, threat appraisals, intolerance of uncertainty and metacognitions [19]. Intrusive thoughts experienced in FoP are similar to pathological worries in GAD, whereby both may differ in content (cancer specific vs. generalized) [17, 20]. Further commonalities in the etiology and maintenance of FoP and GAD include metacognitions and intolerance of uncertainty [19, 21]. There is a gap in literature as to how both constructs are interconnected or how they can be discriminated. One reason for this is the statistical approach chosen by the aforementioned studies, namely the use of sum scores based on self-reported measurements [16–18] Sum scores rely on the assumption of an underlying latent variable (e.g., FoP or GAD) that loads on the items. With this psychometric framework, co-occurrence can only arise in two ways: either (1) due to correlation of the latent variables (i.e. syndromes) with each other or (2) as a consequence of symptoms occurring in both questionnaires, that leads to artificial co-occurrence rates [22].

An alternative way to understand interrelationships between syndromes is the network perspective, in which syndromes emerge from causal relations between symptoms [22]. This results in a complex, dynamic network in which syndromes appear as clusters, i.e., directly interconnected symptoms that maintain each other [22]. Methodologically, this can be implemented via network modelling: it provides the graphical representation of the complex organization of symptoms [22]. A network consists of nodes (i.e., symptoms) and pair interactions between the nodes called edges [22]. If two nodes show a pairwise interaction after controlling for all other nodes in the network (i.e., partial correlations), they are graphically linked by an edge [22]. Network analysis allows us to identify nodes that are most central in the network. In terms of clinical data, these nodes are hypothesized to play a crucial role in maintaining psychopathology, as they are more strongly connected to other nodes in the network than less central nodes [23]. By developing tailored interventions targeting central nodes (i.e., symptoms), treatment efficacy may be improved [24]. Network analysis can also be used to identify nodes of one cluster that have particularly strong or weak associations to a different cluster [25]. In the case of strong associations, we speak of bridge symptoms, that may help to understand co-occurrence in an alternative way [25]. In the case of weak associations, it can theoretically be assumed that these discriminate notably well between the clusters.

So far one study has investigated interrelations between FoP, GAD and depression via network analysis [26]. FoP had only a few weak associations to GAD and depression, while symptoms of GAD and depression were highly connected [26]. As FoP was only assessed on a four-item scale, the insights about interrelations between FoP and GAD are limited. Therefore, our study aimed to gain a better understanding on how FoP and GAD symptoms are interconnected and how both syndromes could be differentiated conceptually. If FoP is a distinct syndrome, it would be fruitful to develop tailored psychotherapeutic interventions. A recent meta-analysis on psychotherapeutic interventions on FoP revealed only small to moderate effects, whereby most interventions were not specifically tailored for FoP [27].

We applied network analyses of GAD and FoP symptoms to a sample of hematological cancer survivors. We (i) investigated the overall network structure of FoP and GAD and (ii) tested a priori hypotheses on specific symptoms in terms of their ability to differentiate both syndromes. Based on the reviewed literature, we assume that intrusive thoughts and worries can be differentiated by their content (cancer related vs. generalized) [17]. This means that, for example, FoP symptoms associated with intrusive thoughts related to cancer (e.g., *Fear of not enduring treatment)* discriminate well between both syndromes. Furthermore, we assume that FoP symptoms related to fear of dying, which is discussed as a central pathomechanism of FoP, also discriminates well between both syndromes [19]. Since various commonalities are discussed in the literature (intolerance to uncertainty, metacognitions), we could not derive specific hypotheses and are exploring the relationships between FoP and GAD exploratively [20].

## Methods

### Participants and data collection

This study is a secondary analysis of a register-based study among hematological cancer survivors. Main purpose of the primary study was to investigate long-term effects of physical, psychological and social domains of quality of life amongst a cross-sectional sample of hematological cancer survivors. Data was collected once from June 2015 to August 2017. Patients were recruited from two German cancer registries and considered eligible if they had a (i) (former) diagnosis of any hematological malignancy (ICD-10: C81-C96), (ii) minimum time since their first diagnosis of ≥ 2.5 years, (iii) minimum age of 18 years at diagnosis and (iv) maximum age of 85 years at study participation. The study was approved by the Ethics Committee of the Medical Faculty at the University of Leipzig (case number: 292–15–24082015) and carried out in accordance with the Declaration of Helsinki. Informed consent was obtained from all individual participants in our study. Further details of the study are described in the published protocol [28] and other outcomes are presented in previous publications [29, 30].

### *Measures*

#### Generalized anxiety disorder questionnaire (GAD-7)

This is a 7-item self-completed questionnaire assessing the symptoms of Generalized Anxiety Disorder [31] based on DSM-IV criteria. Patients rate the frequency of symptoms within the last two weeks (e.g.: “excessive worry regarding various matters”) on a four-point Likert scale ranging from 0 (not at all) to 3 (almost every day), with higher values (sum scores) indicating worse symptomatology. [31]. Cronbach's Alpha was 0.89. The American Society for Clinical Oncology recommends a cut‐off ≥ 10 [1].

#### Fear of progression questionnaire (FoP-Q)

The FoP-Q captures the extent of fear of progression and recurrence in people with medical conditions such as cancer [11]. The questionnaire comprises 5 subscales (“affective reactions”, “partnership/family”, “work”, “loss of autonomy” and “coping”). For the purpose of our study, we used the sub-scale ‘affective reactions’ since this scale contains the core elements of FoP (e.g., “all types of little aches and pain make me anxious”). All 13 items of this sub-scale were rated on a 5-point Likert scale from 0 (never) to 4 (very often), with higher values (sum scores) indicating worse symptomatology. Cronbach's Alpha was 0.93. There is no established cut-off for the affective reactions scale, so we used the reported mean score of the scale from the original study (M = 2.64) [11]. Patients above this value are reported as clinically significant.

### Statistical procedures

All statistical analyses were performed using R Version 3.3.1. We first provide descriptive statistics on sociodemographic and medical characteristics as well as on all symptoms included in the network (means, prevalence of clinical GAD and FoP). Participants were compared with non-participants using Mann–Whitney-U tests (age and time since diagnosis) and Chi-square-tests (gender and type of diagnosis). Participants with missing values in GAD-7 and FoP-Q were excluded from the data set list-wise. Additionally, we compared participants with missing values with participants having complete datasets using Mann–Whitney-U test (age) and Chi-square-tests (gender).

#### Network estimation

The network model consists of nodes (symptoms) and edges (partial correlations between nodes) [32]. Due to non-normality according to a significant Shapiro–Wilk test (p < 0.01), Spearman's correlation was used [33]. To reduce complexity and the risk of false positive edges due to multiple testing [33], we computed a regularized Gaussian graphical model (GGM) using the R package qgraph [33]. The network was visualized using the Fruchterman-Reingold algorithm, where highly connected nodes are located closer together. To identify the most central nodes, we calculated the centrality indices *Expected Influence* (EI), which is defined as the sum of edge-weights a node shares with all other nodes in the network, including negative associations [34].

#### Hypotheses on specificity of symptoms

To test our hypotheses on specific symptoms in terms of their ability to differentiate between the syndromes we used the bridge centrality metric from the R package network tools [25]. We calculated the *Bridge Expected Influence* (BEI) for each node, which is defined as the sum of all edge weights a node shares with the nodes from the other community (i.e., syndrome) [25]. A low BEI means few and weak associations, which can indicate a node’s distinctive characteristics, whereas a high BEI means many and strong associations, which can indicate potential co-occurrence pathways or common characteristics.

Based on the reviewed literature, we hypothesized that intrusive thoughts and worries could be differentiated by their content (cancer related vs. generalized) [20]. We further assumed that death-anxiety is a FoP specific characteristic, as it is discussed as a significant pathomechanism [19]. We then selected items from the FoP and GAD questionnaires in regard to these assumptions. Among the GAD items, we selected GAD-3 (*Worry about many things*) as it relates to generalized worries. Among the FoP items, we selected items that were related to cancer specific intrusive thoughts i.e., FoP-1 (*Disease progression),* FoP-7 *(Fear not to endure treatment),* FoP-11* (Severe treatment),* FoP-12* (Medical follow-ups)* and FoP 13 *(Harm by medication).* In terms of fear of dying we selected FoP-9 (*Dying*). Our hypotheses were first tested descriptively and could be considered confirmed if the selected items showed the lowest BEI among all items of the respective questionnaire. In a second step, all hypotheses were statistically investigated: we tested whether the BEI of our a priori selected items were significantly smaller than all other items within both syndromes via bootstrap difference tests.

As various commonalities between FoP and GAD are discussed in the literature (e.g., metacognitions and intolerance of uncertainty) [19, 21], we could not derive specific hypotheses and are exploring the relationships between FoP and GAD exploratively. In doing so, we examine items that have a high BEI.

#### Sensitivity analysis

To ensure the robustness of our findings, sensitivity analyses were performed for the following parameters: edge weights, node centrality indices and bridge centrality indices. In order to assess the reliability of the edge weights and node (bridge-)centrality indices, we computed stability coefficients using the R package network tools. As a threshold to indicate stability, the coefficients should be above 0.5 [33]. Based on the bootstrapped subsamples, confidence intervals were calculated for all parameters. This allows testing for statistical differences, whereas the expected significance level for 1000 bootstrap samples is p = 0.05.

## Results

### Participants

Out of 2001 eligible survivors, 922 (46%) participated. Compared to patients who did not respond or refused (non-participants), participants were younger (Z =  − 3.5, p = 0.001; total mean difference = 1.5 years), but did not differ in type of diagnosis, time since diagnosis or gender (Table 1). 13% had to be removed from the analyses due to missing data, resulting in 805 cases. There were no differences in age and gender between participants with full data sets compared to participants with missing data (p > 0.05). 53% of participants were female and the average age was 63.9 years. The mean scores for the GAD-7 and the FoP-Q were 3.5 and 12.6 (Table 2). According to the recommended cut-off ≥ 10, the prevalence for clinical GAD levels in our sample was 6.8%. For clinical FoP levels the prevalence was 4.2%.

**Table 1:** Sample characteristics among long-term survivors of hematological malignancies

Participants *N* = 922	
N (valid %)	
Sociodemographic	
Gender (Female)	527 (53)
Age, *M (SD)*	63.9 (13.4)
In partnership	655 (74)
Medical	
Cancer type according to ICD-10^a^	
Hodgkin lymphoma (C81)	101 (11)
Follicular lymphoma (C82)	123 (13)
Non-follicular lymphoma (C83)^b^	247 (27)
Other ttypes of lymphoma (C85)	59 (6)
MM/MPCN (C90)^c^	118 (13)
Lymphoid leukemia (C91)^d^	140 (15)
Myeloid Leukemia (C92)^e^	95 (10)
Others	39 (4)
Years since diagnosis, *M (SD)*	9.1 (4.2)
In remission	634 (73)
History of relapse	201 (24)
Second tumor^f^	155 (17)
Treatment^g^	
Chemotherapy	722 (79)
Radiotherapy	391 (43)
Anti-body therapy	198 (22)
Surgery	151 (17)
SCT	244 (27)

*M*  mean, *SD*  standard deviation, MM/MPCN, Multiple myeloma and malignant plasma cell neoplasms, SCT, stem cell transplantation, ^a^International Statistical Classification of Diseases, 10th revision, ^b^Mostly B-cell lymphoma (52%), ^c^Mostly multiple myeloma (95%), ^d^Mostly chronic (76%), ^e^Mostly acute (69%), ^f^Before or after hematological malignancy, ^g^All treatment related to the hematological malignancy, combinations possible

**Table 2:** Descriptive statistics and BEI for GAD-7 and FoP-Q items

Items	*M (SD)*	*BEI*
GAD-7		
1. Nervous	.51 (.70)	.27
2. Uncontrollable Worry	.41 (.68)	.11
3. Worry about many things	.57 (.74)	.12
4. Unable to relax	.68 (.81)	.17
5. Restlessness	.37 (.67)	.08
6. Annoyance	.60 (.70)	.23
7. Fear of awful events	.40 (.70)	.43
Sum score	3.52 (3.91)	
FoP-Q		
1. Disease progression (FoP-1)	1.39 (1.04)	.00
2. Physical reaction of anxiety (FoP-2)	1.02 (1.07)	.25
3. Attack by anxiety (FoP-3)	.85 (.98)	.13
4. Impairment of joy (FoP-4)	.78 (1.01)	.14
5. Sleep disturbances (FoP-5)	.78 (1.01)	.16
6. Hypervigilance (FoP-6)	.84 (1.05)	.19
7. Fear not to endure treatment (FoP-7)	.57 (.92)	.02
8. Pain (FoP-8)	1.09 (1.10)	.03
9. Dying (FoP-9)	1.02 (1.12)	.05
10. Irritability (FoP-10)	.59 (.93)	.40
11. Severe treatment (FoP-11)	1.02 (1.09)	.03
12. Medical follow-ups (FoP-12)	1.28 (1.21)	.01
13. Harm by medication (FoP-13)	1.34 (1.25)	.02
Sum score	12.56 (10.28)	

*M* Mean, *SD* standard deviation, *BEI* Bridge Expected Influence

### Network estimation

**Fig. 1:**
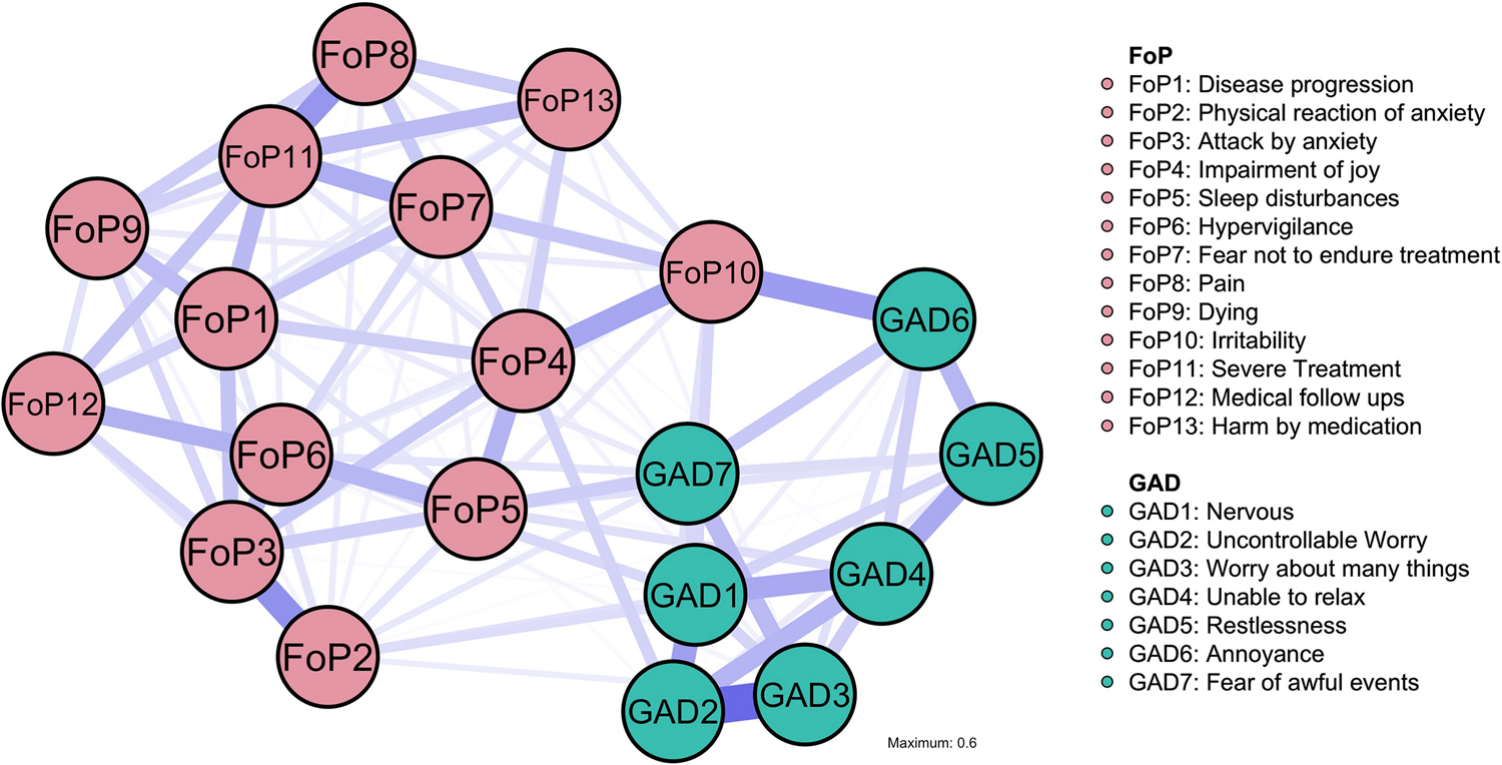
Network estimation of GAD and FoP symptoms in hematological cancer survivors

Of 190 possible edges, 103 (54%) had an absolute edge weight (i.e., partial correlation coefficient) above zero (Fig. 1). Similar to the correlation coefficient, the partial correlation coefficient also has a value in the range of -1 to 1. Across all items, the mean edge weight was .049. Among GAD and FoP items, the mean edge weights were .13 and .07, respectively. The mean edge weight between items of both questionnaires was .01. This is understandable as the items within the questionnaires were more correlated with each other than between syndromes, where the mean partial correlation was close to 0, indicating that there is only a marginal linear relationship. The strongest edge-weight in the network was between GAD-2 (*uncontrollable worry*) and GAD-3 (*worry about many things*). The strongest edge-weight between both constructs was found between the items GAD-6 (*Annoyance*) and FoP-10 (*Irritability*).

#### Testing hypotheses of GAD symptoms

Testing our a priori formed hypothesis for distinct characteristics within both constructs we found GAD-3 (*worry about many things*) to be among the three nodes with the smallest BEI. Secondly, our pairwise difference tests revealed only two out six statistically relevant differences (Table 3). This confirms our assumption on GAD-3 descriptively, but not statistically.

**Table 3:** Statistical validation of the hypotheses on distinct symptoms for each construct

Testing of hypothesis regarding GAD symptoms								
	GAD-1	GAD-2	GAD-4	GAD-5	GAD-6	GAD-7		
GAD-3	< .05*					< .05*		
Testing of hypotheses regarding FoP symptoms								
	FoP-2	FoP-3	FoP-4	FoP-5	FoP-6	FoP-8	FoP-9	FoP-10
FoP-1	< .05*	< .05*	< .05*	< .05*	< .05*			< .05*
FoP-7	< .05*				< .05*			< .05*
FoP-11	< .05*				< .05*			< .05*
FoP-12	< .05*	< .05*	< .05*	< .05*	< .05*			< .05*
FoP-13	< .05*	< .05*	< .05*	< .05*	< .05*			< .05*
FoP-9	< .05*	< .05*		< .05*	< .05*			< .05*

Notes *p*-values refer to pairwise difference tests of BEI of the respective items

#### Testing hypotheses of FoP symptoms

In line with our hypothesis on distinct characteristics of FoP symptoms related to cancer specific intrusive thoughts, FoP-1 (*Disease progression*), FoP-7 (*Fear not to endure treatment*), FoP-11 (*Severe treatment*), FoP-12 (*Medical follow-ups*), FoP-13 (*Harm by Medication*) had the smallest BEI amongst all FoP items (bridge centrality indices can be seen in figure S1). Statistically, no differences were found between all a priori selected items and FoP-8 (*Pain*) (Table 1). The item FoP-9 (*Dying*) was also amongst the items having a particularly small BEI. This confirms our assumption on FoP items descriptively but not statistically.

Items with the highest BEI indicating potential co-occurrence pathways between the communities were GAD-7 (*Fear of awful events*) and FoP-10 (*Irritability*) (Table 2). Both items were significantly different from 90% of all items in the network (p < .05).

### Sensitivity analysis

To ensure the robustness of our findings, Stability-Coefficients (SC) were estimated for the following parameters: edge weights, centrality indices and bridge centrality indices. Above a threshold of SC > 0.5, the findings can be considered stable and interpretable. The SC for the edge weights was .75, indicating very high stability. For the (bridge-) centrality measures, the SCs for both EI and BEI were 0.75, which also provides high stability.

## Discussion

This study examined the relationship between FoP and GAD symptoms among cancer patients via network analysis. Mean associations were higher within each syndrome than between them, which was also reflected in the separate clustering of the items in the network.

Our results are consistent with a recent study that also found only few associations between FoP and GAD symptoms in the network [26]. Since only four items were used to capture FoP in the previous study, the transferability of those findings to our data is limited. Nevertheless, it supports the assumption that FoP is conceptually different from GAD. Previous works examining the interrelationship between FoP and GAD have used the psychometric framework of the latent variable model (i.e., using sum-scores, categorial diagnoses), which can only provide limited insight into the interrelations between FoP and GAD [22]. By applying the network perspective, we were able to investigate the interconnections between both syndromes at the symptom level. Regarding our first hypothesis concerning conceptual differentiation based on the content of intrusive thoughts and worries, we found the GAD symptom *worry about many things* among the symptoms that were least associated with FoP symptoms. This assumption could be confirmed descriptively but not statistically, as there were symptoms with few associations that were not pre-selected and for which there were no significant differences (i.e., *uncontrollable worry, unable to relax, restlessness and annoyance).* With respect to cancer-related worries, our assumption was also descriptively confirmed: pre-selected symptoms (e.g., *disease progression* or *medical follow-ups*) had the weakest associations with GAD. The same applied to our second hypothesis: we found the FoP symptom *dying* among least associated symptoms with GAD. Besides our pre-selected symptoms, we also found that worries related to *Pain* were weakly associated with GAD symptoms as well. A potential explanation might be that fear of pain is one the most frequently reported fears among cancer patients [35]. We identified *Fear of awful events* (GAD) and *Irritability* (FoP) to be the symptoms most associated with the other syndrome. *Irritability* shared a strong association with the GAD symptom *Annoyance,* which we attributed to a characteristic both syndromes have in common as a result of persistent occupation with worries. The GAD symptom *Fear of awful events* was strongly connected with the FoP symptoms *Attack by anxiety, Irritability and Hypervigilance*. This seems plausible considering that cancer patients have a real fear e.g., of recurrence or new impairments as a consequence of invasive treatments. It is also noticeable that there are only associations with symptoms that are not specific to cancer. One possible explanation would be that theoretical models on the etiology of GAD emphasize the role of hyperreactivity towards negative events [21]. This would indicate that the content of the worries differs between FoP and GAD, but the way they are dealt with and reacted to is similar. Additionally, the association could be explained by intolerance of uncertainty, which is also discussed as a significant pathomechanism for both syndromes [19, 21].

### Implications for diagnostics and treatment in oncological settings

Our findings support the assumption of FoP as a distinct syndrome, which implies a need to work on the development of tailored interventions targeting central symptoms [27]. Central symptoms which could be targeted are *attack by anxiety, impairment of joy, irritability* and *severe treatment.* Regarding differential diagnostics in cancer patients, it seems helpful to explore the content of worries in more detail. It also seems reasonable to systematically screen cancer patients for FoP.

### Strength and limitations

A particular strength of the present work is our large sample size which enabled sufficient test power for network analyses. We defined a priori hypotheses based on current research in order to be as theory-driven as possible despite the explorative character of our study. The hypotheses were tested descriptively, but also statistically validated. A major limitation was that the cross-sectional data does not allow any conclusions about causal relationships between symptoms. Characteristics that predispose people to develop FoP or GAD cannot be displayed. Moreover, our sample is specific, as it includes exclusively hematological cancer survivors and is not representative for other types of cancer. The factor-analytic approach to questionnaire construction may also have influenced the symptom clustering in our network [33]. Even though the GAD-7 and FoP-Q are psychometrically validated, these complex constructs are captured by relatively few symptoms and thus many potentially relevant aspects are lost. This issue may be even more relevant in our study since we only used one subscale from the FoP-Q. For future studies, it might be fruitful to use extended questionnaires with more detailed items to explore the dynamics between FoP and GAD in a more profound way. For example, metacognitions and intolerance of anxiety seem to play crucial roles in the etiology and maintenance of FoP and GAD. This should be considered when selecting instruments in future studies [7, 36].

## Conclusion

Using a novel statistical approach, we were able to show that the content of worries (cancer related vs. generalized) and fear of dying are certain characteristics which may be relevant aspects to differentiate between FoP and GAD among cancer survivors. Nevertheless, there is also indication of common pathomechanisms, which appear as associations between both syndromes. Future studies are needed to verify our hypotheses-driven but exploratory findings .

## Supplementary Information

Below is the link to the electronic supplementary material.

Supplementary file1 (DOCX 185 KB)

## Data Availability

The datasets analyzed during the current study are available from the corresponding author on reasonable request.
