# Supplementary material for: Interconnectivity of fear of progression and generalized anxiety – Network analysis among a sample of hematological cancer survivors
Source: Support Care Cancer. 2023 Mar 28;31(4):238. doi: 10.1007/s00520-023-07701-x (PMC10042941; doi:10.1007/s00520-023-07701-x)
Supplement: Supplementary file 1 — Supplementary file1 (DOCX 185 KB) [file 520_2023_7701_MOESM1_ESM.docx]

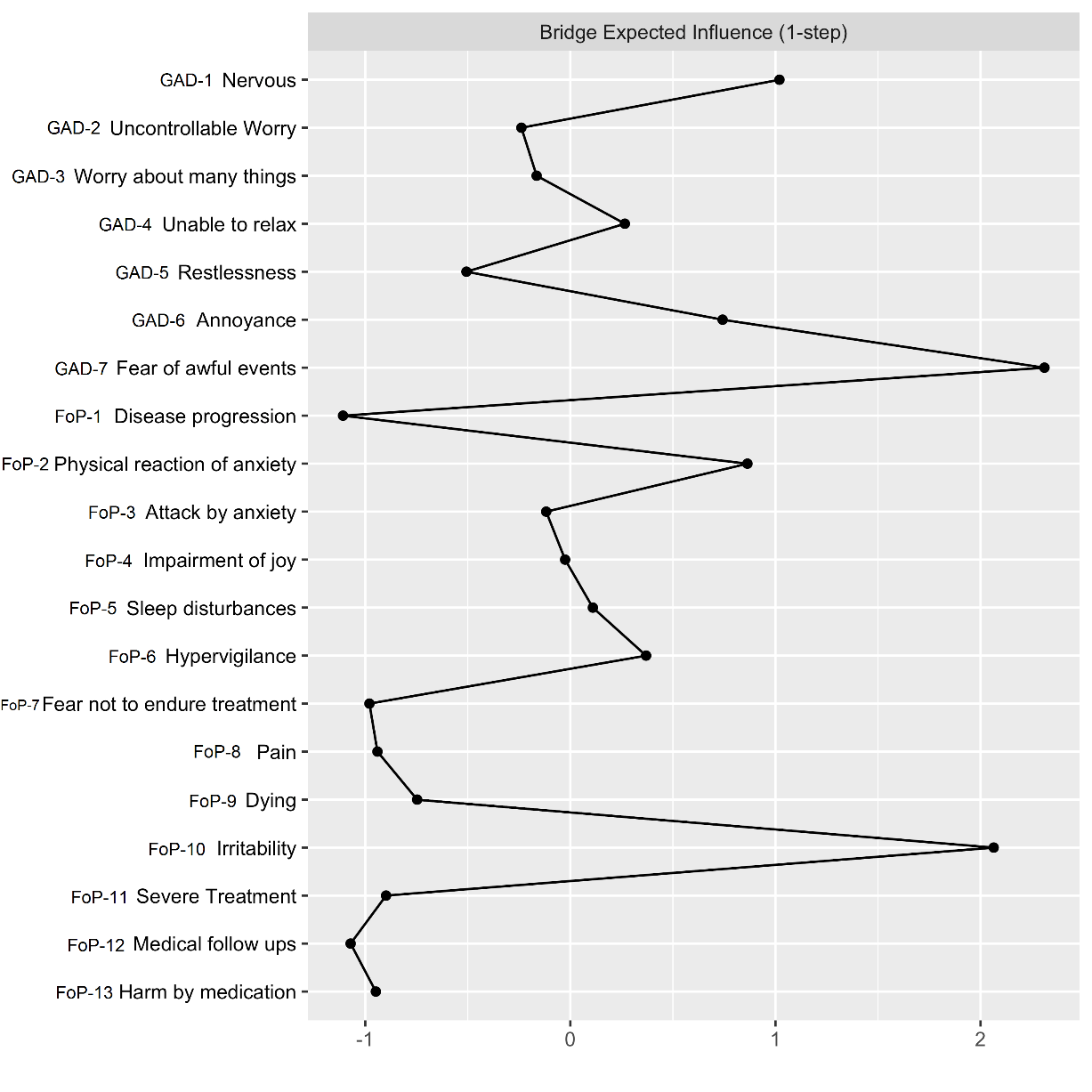


**Figure S2** Centrality plot for the association network depicting the bridge expected influence (BEI) of each node (GAD and FoP items). Higher values indicate more associations with the other construct.
